# Supplementary material for: Equity of travel required to access first definitive surgery for liver or stomach cancer in New Zealand
Source: PLoS One. 2022 Aug 11;17(8):e0269593. doi: 10.1371/journal.pone.0269593 (PMC9371338; doi:10.1371/journal.pone.0269593)
Supplement: S1 File — (DOCX) [file pone.0269593.s001.docx]

# Supplementary Material

**Supplementary Material 1:** Demographic and patient characteristics of all Māori and European patients diagnosed with liver and stomach cancer between 2017-2019.

|  | **Liver** | | | | | | **Stomach** | | | | | |
| --- | --- | --- | --- | --- | --- | --- | --- | --- | --- | --- | --- | --- |
|  | **Māori** | | | **European** | | | **Māori** | | | **European** | | |
|  | n | % | Age Std. % | n | % | Age Std. % | n | % | Age Std. % | n | % | Age Std. % |
| Total | 866 | - | - | 2,460 | - | - | 953 | - | - | 3,192 | - | - |
| Age (years) |  |  |  |  |  |  |  |  |  |  |  |  |
| <50 | 140 | 16% | - | 128 | 5% | - | 216 | 23% | - | 195 | 6% | - |
| 50-64 | 429 | 50% | - | 718 | 29% | - | 343 | 36% | - | 696 | 22% | - |
| 65-74 | 186 | 21% | - | 683 | 28% | - | 227 | 24% | - | 883 | 28% | - |
| 75+ | 111 | 13% | - | 931 | 38% | - | 167 | 18% | - | 1,418 | 44% | - |
| Sex |  |  |  |  |  |  |  |  |  |  |  |  |
| Female | 226 | 26% | 27% | 830 | 34% | 31% | 416 | 44% | 44% | 1,042 | 33% | 32% |
| Male | 640 | 74% | 73% | 1,630 | 66% | 69% | 537 | 56% | 56% | 2,150 | 67% | 68% |
| Deprivation (NZDep Decile) |  |  |  |  |  |  |  |  |  |  |  |  |
| 1-2 (least deprived) | 51 | 6% | 6% | 372 | 16% | 16% | 40 | 4% | 4% | 487 | 16% | 16% |
| 3-4 | 61 | 7% | 7% | 429 | 18% | 18% | 80 | 9% | 8% | 563 | 18% | 18% |
| 5-6 | 105 | 12% | 12% | 525 | 22% | 21% | 118 | 13% | 12% | 668 | 22% | 21% |
| 7-8 | 194 | 23% | 23% | 580 | 24% | 23% | 208 | 22% | 22% | 778 | 25% | 24% |
| 9-10 (most deprived) | 439 | 52% | 51% | 487 | 20% | 19% | 485 | 52% | 51% | 578 | 19% | 16% |
| Rurality (URPC Category) |  |  |  |  |  |  |  |  |  |  |  |  |
| Urban | 582 | 69% | 67% | 1,731 | 72% | 72% | 605 | 65% | 64% | 2,179 | 71% | 68% |
| Independent Urban | 150 | 18% | 18% | 399 | 17% | 14% | 161 | 17% | 17% | 493 | 16% | 14% |
| Rural | 117 | 14% | 14% | 260 | 11% | 11% | 164 | 18% | 17% | 399 | 13% | 14% |
| Stage (SEER Category) |  |  |  |  |  |  |  |  |  |  |  |  |
| Local | 89 | 10% | 10% | 178 | 7% | 10% | 107 | 11% | 11% | 210 | 7% | 7% |
| Regional | 23 | 3% | 2% | 87 | 4% | 4% | 163 | 17% | 17% | 401 | 13% | 14% |
| Advanced | 188 | 22% | 22% | 588 | 24% | 25% | 353 | 37% | 37% | 1,031 | 32% | 37% |
| Unstaged | 566 | 65% | 65% | 1,607 | 65% | 61% | 330 | 35% | 34% | 1,550 | 49% | 42% |
| Comorbidity (C3 Index Category) |  |  |  |  |  |  |  |  |  |  |  |  |
| 0 | 203 | 23% | 24% | 819 | 33% | 37% | 493 | 52% | 52% | 1,654 | 52% | 62% |
| 1 | 250 | 29% | 28% | 534 | 22% | 23% | 129 | 14% | 14% | 463 | 15% | 14% |
| 2 | 158 | 18% | 19% | 424 | 17% | 18% | 98 | 10% | 10% | 381 | 12% | 10% |
| 3 | 255 | 29% | 29% | 683 | 28% | 22% | 233 | 24% | 24% | 694 | 22% | 13% |

C3 index category: ’0’ (C3 Index score < =0), ‘1’ (0 < score < =1), ‘2’ (1 < score < =2) and ‘3’ (score >2).

**Supplementary Material 2:** Hospital volume categorisation for each of the four included procedures: a) minor hepatectomy, b) liver ablation, c) partial gastrectomies, and d) endoscopic injections. The number of procedures per year were determined by dividing the total number of procedures that occurred over the 13-year period by 13.

| a) Minor Hepatectomy | | | |
| --- | --- | --- | --- |
| **Facility Name** | **#/year** | **Category** | **Rationale** |
| Auckland City Hospital | 50 | High | ~1 per week |
| Christchurch Hospital | 22.69 | Medium | ~1 per 1-2 months |
| North Shore Hospital | 11.69 | Medium | ~1 per 1-2 months |
| Dunedin Hospital | 10.23 | Medium | ~1 per 1-2 months |
| Wellington Hospital | 9.85 | Medium | ~1 per 1-2 months |
| Waikato Hospital | 9 | Medium | ~1 per 1-2 months |
| Tauranga Hospital | 1 | Low | <=1 per year |
| Southern Cross Hospital Wellington | 0.92 | Low | <=1 per year |
| Gisborne Hospital | 0.85 | Low | <=1 per year |
| Palmerston North Hospital | 0.85 | Low | <=1 per year |
| Southland Hospital | 0.85 | Low | <=1 per year |
| Whangarei Hospital | 0.77 | Low | <=1 per year |
| Nelson Hospital | 0.54 | Low | <=1 per year |
| Hutt Hospital | 0.46 | Low | <=1 per year |
| Middlemore Hospital | 0.46 | Low | <=1 per year |
| Manukau SuperClinic | 0.38 | Low | <=1 per year |
| Rotorua Hospital | 0.31 | Low | <=1 per year |
| Whanganui Hospital | 0.31 | Low | <=1 per year |
| Hawkes Bay Hospital | 0.23 | Low | <=1 per year |
| St Georges Hospital | 0.15 | Low | <=1 per year |
| Timaru Hospital | 0.15 | Low | <=1 per year |
| Anglesea Hospital | 0.08 | Low | <=1 per year |
| Ashburton Hospital | 0.08 | Low | <=1 per year |
| Christchurch Womens Hospital | 0.08 | Low | <=1 per year |
| Kenepuru Community Hospital | 0.08 | Low | <=1 per year |
| Southern Cross Hospital Christchurch | 0.08 | Low | <=1 per year |
| Taranaki Base Hospital | 0.08 | Low | <=1 per year |
| Te Nikau Grey Hospital | 0.08 | Low | <=1 per year |
| Wairau Hospital | 0.08 | Low | <=1 per year |

| b) Liver Ablation | | | | |
| --- | --- | --- | --- | --- |
| **Facility Name** | **#/year** | **Category** | **Rationale** |  |
| Auckland City Hospital | 77 | High | ~1 per 1-2 weeks |  |
| Waikato Hospital | 34.69 | High | ~1 per 1-2 weeks |  |
| Christchurch Hospital | 22.46 | High | ~1 per 1-2 weeks |  |
| Wellington Hospital | 15.23 | Medium-High | ~1 per month |  |
| Hutt Hospital | 10.54 | Medium-High | ~1 per month |  |
| Dunedin Hospital | 3.85 | Medium-Low | ~1 per 3 months |  |
| Middlemore Hospital | 3.38 | Medium-Low | ~1 per 3 months |  |
| Tauranga Hospital | 1.92 | Low | ~1 per >6 months |  |
| North Shore Hospital | 1.62 | Low | ~1 per >6 months |  |
| Hawkes Bay Hospital | 0.92 | Low | ~1 per >6 months |  |
| Nelson Hospital | 0.46 | Low | ~1 per >6 months |  |
| Palmerston North Hospital | 0.46 | Low | ~1 per >6 months |  |
| Wakefield Hospital | 0.38 | Low | ~1 per >6 months |  |
| Southern Cross Hospital Wellington | 0.23 | Low | ~1 per >6 months |  |
| Braemar Hospital | 0.15 | Low | ~1 per >6 months |  |
| Christchurch Womens Hospital | 0.15 | Low | ~1 per >6 months |  |
| Taranaki Base Hospital | 0.15 | Low | ~1 per >6 months |  |
| Manukau SuperClinic | 0.08 | Low | ~1 per >6 months |  |
| Mercy Integrated Hospital | 0.08 | Low | ~1 per >6 months |  |
| Rotorua Hospital | 0.08 | Low | ~1 per >6 months |  |
| Whanganui Hospital | 0.08 | Low | ~1 per >6 months |  |
| Whangarei Hospital | 0.08 | Low | ~1 per >6 months |  |

| c) Partial Gastrectomy | | | |
| --- | --- | --- | --- |
| **Facility Name** | **#/year** | **Category** | **Rationale** |
| Auckland City Hospital | 17.62 | High | ~1 per month |
| North Shore Hospital | 17.38 | High | ~1 per month |
| Waikato Hospital | 15.54 | High | ~1 per month |
| Christchurch Hospital | 14.92 | High | ~1 per month |
| Manukau SuperClinic | 11.31 | High | ~1 per month |
| Palmerston North Hospital | 11.23 | High | ~1 per month |
| Middlemore Hospital | 8.69 | Medium-High | ~1 per 1-3 months |
| Dunedin Hospital | 7.38 | Medium-High | ~1 per 1-3 months |
| Wellington Hospital | 6.15 | Medium-High | ~1 per 1-3 months |
| Whangarei Hospital | 5.85 | Medium-High | ~1 per 1-3 months |
| Tauranga Hospital | 5.69 | Medium-High | ~1 per 1-3 months |
| Hutt Hospital | 4.08 | Medium-High | ~1 per 1-3 months |
| Hawkes Bay Hospital | 3.31 | Medium-High | ~1 per 1-3 months |
| Southland Hospital | 3.08 | Medium-High | ~1 per 1-3 months |
| Taranaki Base Hospital | 2.38 | Medium-Low | ~1 per 6 months |
| Timaru Hospital | 2.23 | Medium-Low | ~1 per 6 months |
| Rotorua Hospital | 2.15 | Medium-Low | ~1 per 6 months |
| Ormiston Surgical & Endoscopy Limited | 2 | Medium-Low | ~1 per 6 months |
| Ascot Integrated Hospital | 1.85 | Medium-Low | ~1 per 6 months |
| Whanganui Hospital | 1.85 | Medium-Low | ~1 per 6 months |
| Nelson Hospital | 1.77 | Medium-Low | ~1 per 6 months |
| St Georges Hospital | 1.54 | Medium-Low | ~1 per 6 months |
| Gisborne Hospital | 1.31 | Medium-Low | ~1 per 6 months |
| Southern Cross Hospital Brightside | 0.54 | Low | <=1 per year |
| Whakatane Hospital | 0.54 | Low | <=1 per year |
| Wairarapa Hospital | 0.46 | Low | <=1 per year |
| Wairau Hospital | 0.31 | Low | <=1 per year |
| Elective Surgery Centre - Waitemata DHB | 0.23 | Low | <=1 per year |
| Southern Cross Hospital Christchurch | 0.23 | Low | <=1 per year |
| Te Nikau Grey Hospital | 0.23 | Low | <=1 per year |
| Wakefield Hospital | 0.23 | Low | <=1 per year |
| Thames Hospital | 0.15 | Low | <=1 per year |
| Ashburton Hospital | 0.08 | Low | <=1 per year |
| Auckland City Surgical Services | 0.08 | Low | <=1 per year |
| Braemar Hospital | 0.08 | Low | <=1 per year |
| Kensington Hospital | 0.08 | Low | <=1 per year |
| Southern Cross Hospital Wellington | 0.08 | Low | <=1 per year |

| d) Endoscopic Injection | | | |
| --- | --- | --- | --- |
| **Facility Name** | **#/year** | **Category** | **Rationale** |
| Auckland City Hospital | 8.08 | High | ~1 per 1-2 months |
| Waikato Hospital | 7.92 | High | ~1 per 1-2 months |
| Ascot Integrated Hospital | 7.85 | High | ~1 per 1-2 months |
| North Shore Hospital | 6.69 | High | ~1 per 1-2 months |
| Christchurch Hospital | 6.54 | High | ~1 per 1-2 months |
| Tauranga Hospital | 4.31 | Medium | ~1 per 3 months |
| Hawkes Bay Hospital | 3.23 | Medium | ~1 per 3 months |
| Middlemore Hospital | 2.85 | Medium | ~1 per 3 months |
| Wellington Hospital | 2.69 | Medium | ~1 per 3 months |
| Palmerston North Hospital | 2.54 | Medium | ~1 per 3 months |
| Whangarei Hospital | 2.23 | Medium | ~1 per 3 months |
| Dunedin Hospital | 1.46 | Low | <=1 per year |
| Taranaki Base Hospital | 1.46 | Low | <=1 per year |
| Gisborne Hospital | 1.31 | Low | <=1 per year |
| Rotorua Hospital | 1.15 | Low | <=1 per year |
| Whanganui Hospital | 1.08 | Low | <=1 per year |
| Nelson Hospital | 1 | Low | <=1 per year |
| Hutt Hospital | 0.54 | Low | <=1 per year |
| Timaru Hospital | 0.54 | Low | <=1 per year |
| Southland Hospital | 0.46 | Low | <=1 per year |
| Whakatane Hospital | 0.46 | Low | <=1 per year |
| Te Nikau Grey Hospital | 0.15 | Low | <=1 per year |
| Wairarapa Hospital | 0.15 | Low | <=1 per year |
| Manukau SuperClinic | 0.08 | Low | <=1 per year |
| Mercy Integrated Hospital | 0.08 | Low | <=1 per year |
| Southern Cross Hospital Christchurch | 0.08 | Low | <=1 per year |
| Wairau Hospital | 0.08 | Low | <=1 per year |
